# Symmetry and symmetry breaking in cancer: a foundational approach to the cancer problem

**DOI:** 10.18632/oncotarget.22939

**Published:** 2017-12-05

**Authors:** J. James Frost, Kenneth J. Pienta, Donald S. Coffey

**Affiliations:** ^1^ Department of Radiology, Johns Hopkins University School of Medicine, Baltimore, MD, USA; ^2^ James Buchanan Brady Urological Institute at the Johns Hopkins University School of Medicine, Baltimore, MD, USA; ^3^ Department of Medical Oncology, Johns Hopkins School of Medicine and Sidney Kimmel Comprehensive Cancer Center, Baltimore, MD, USA; ^4^ Department of Pharmacology and Molecular Sciences, Johns Hopkins School of Medicine, Baltimore, MD, USA

**Keywords:** cancer, symmetry, symmetry-breaking, complexity, scale-free

## Abstract

Symmetry and symmetry breaking concepts from physics and biology are applied to the problem of cancer. Three categories of symmetry breaking in cancer are examined: combinatorial, geometric, and functional. Within these categories, symmetry breaking is examined for relevant cancer features, including epithelial-mesenchymal transition (EMT); tumor heterogeneity; tensegrity; fractal geometric and information structure; functional interaction networks; and network stabilizability and attack tolerance. The new cancer symmetry concepts are relevant to homeostasis loss in cancer and to its origin, spread, treatment and resistance. Symmetry and symmetry breaking could provide a new way of thinking and a pathway to a solution of the cancer problem.

## THE CANCER PROBLEM

Cancer remains unsolved. It is a problem that has resisted solution for centuries, in spite of the application of immense bodies of knowledge from diverse fields, including molecular biology, biochemistry, pharmacology and physics [1]. Cancer functions as a complex system that enables an unrelenting adaptability to a large range of environmental changes, including alterations in nutrients, oxygen, pH, temperature, and treatment with interventional agents. New ways of thinking about the cancer problem are still needed.

A still prevalent cancer paradigm from the time of Beale (1860) and Boveri (1902) is that cancer is associated with structural abnormalities, from tissue and cell structure to DNA organization [2–6]. Cancer also manifests many structure-independent functional abnormalities, including dysregulated metabolism, epigenetic alterations, and transcription factor dysfunction [7–11]. Information storage at the level of DNA is another level of disruption in cancer that includes mutations and translocations [12, 13]. In cancer, the transformation from the well-regulated homeostasis of the normal cell to the chaotic, uncoordinated cancer state has been described as a phase transition that involves extensive change in structure, function and information [14]. The cancer transition involves alterations across all scales, including DNA, the cell, and communication among cancer and cancer-supporting cells. Therein lies the complexity of cancer: the myriad of innumerable interactions among the individual molecular agents within the cancer cell, among other cells types in the cancer microenvironment and across the organ systems of the host.

This cancer feature is similar to a problem in physics that is simple in its description, but has withstood exact solution since the time of Newton: the three-body problem [15]. When two bodies, such as the sun and the Earth, interact dynamically through gravitation, their movement in space can be computed with ease. Adding just one additional body, for example the Moon, introduces a level of complexity and nonlinearity to the problem such that even an approximate solution is computed with difficulty. As more bodies are added, the problem difficulty increases in exponential computation time. Therefore, it is not surprising that cancer biology remains at an impasse as it confronts a problem that involves a practicably uncountable number of interacting cell agents. In the three-body or multi-body problems of physics, major simplification is introduced by consideration of symmetry, resulting in new solutions that can be classified based on their symmetry features [16–18]. Analogously, the problem of cancer may be better understood and rendered more tractable by analysis of its symmetry features. The field of biology is filled with underappreciated examples of symmetry and symmetry breaking. Normal cell and tissue function is a result of tightly controlled maintenance of symmetry and concomitant symmetry breaking when required.

## SYMMETRY IN PHYSICS AND BIOLOGY

Symmetry is an elemental feature of space and time that underlies the geometric and dynamical properties of the observable universe [19, 20]. Symmetry is information: information about that which remains unchanged or, in physics nomenclature, is invariant when an operation on a system is carried out. Geometric symmetries are best known and are observed throughout the natural world. The snowflake is a well-known example; it possesses 6-fold rotational and 12-fold reflection symmetry. In the example of rotational symmetry, the results of an experiment performed in a spaceship far away from gravitational or other fields don’t have to be replicated for every direction the laboratory is oriented. Physical symmetries also extend to the known forces and particles in the universe, such as the existence of the negatively charged electron and the positively charged, but equal mass, positron. The search for the Higgs boson and its recent discovery was the result of employing symmetry principles [21]. At an information level, symmetry permits a more compact system description and simplifies computational problems.

The complement of symmetry is symmetry breaking [22–24]. Broken symmetry can result from explicit or from spontaneous symmetry breaking. Explicit symmetry breaking is the most familiar and occurs, for example, when the side of an apple is cut off or when an egg is dropped on the floor and shatters. Spontaneous symmetry breaking is conceptually more difficult, but occurs throughout the universe with regularity. When a magnet is heated above a certain temperature called the Curie point the magnetization is lost as all the individual magnetic particles assume random orientations in a symmetric pattern. When the temperature is reduced below the Curie point in the spaceship laboratory far from the Earth’s magnetic field the individual particles re-coalesce into a magnet, but the north-south orientation of the magnet is not predictable— this is an example of spontaneous symmetry breaking. Similarly, when water freezes, the axis orientation of the ice crystals is random. In the language of physics, at the exact transition point of instability the lowest energy solution that respects the initial symmetry ceases to be the lowest energy solution and a new, but asymmetric solution becomes the new low energy solution. In biology, an organism utilizes symmetry breaking along well-defined axes for functional diversification on every scale, from molecular assemblies, to subcellular structures, to cell types themselves, to tissue architecture [25]. Normal cell and tissue function is a result of tightly controlled maintenance of symmetry and symmetry breaking when required, for example during development.

In physics, symmetry is most often preserved and therefore forms a basis for the search of fundamental particles and forces. In biology symmetry breaking occurs continuously and indeed, is a condition for life [26, 27]. This symmetry breaking is, however, always incomplete: completely broken symmetry is complete disorder, which could not sustain life. Correspondingly, perfect order and global symmetry would also be incompatible with life. The informational content of a perfectly symmetric system is inadequate for the complex functions of life. Life exists in the intermediate realm between order and disorder. Cancer may be a state of broken symmetry beyond that of the normal homeostasis and the controlled system of sustainable life.

## SYMMETRY BREAKING

The complexity of any system can be described and quantified by three components: combinatorial, geometric and functional [27]. Each of these components can be characterized by their symmetries, which can then be applied to understanding specific features of cancer. While each component can be described independently, it is important to note that biology makes use of all three together.

### Combinatorial symmetry breaking

Combinatorial complexity at the cellular level refers to the number of configurations—genetic or phenotypic—in which cells can be exchanged while maintaining the overall functional invariance of the system [27]. In most normal tissues, cell division results in identical or nearly identical daughter pairs. In cancer, cell division is often an asymmetric process that can be thought of as a series of symmetry breaking events. When occurring over many cell divisions a population of cancer cells displays tumor heterogeneity where a high number of cells have slightly different genetic and phenotypic states. This is one key feature that enables the profound adaptability of cancer. A complete picture of cancer’s combinatorial complexity and symmetry also requires consideration of the cells in the microenvironment, including immune cells, tumor macrophages and many others.

Combinatorial complexity is quantified as:

K_c_ = log(N!/n_i_!) for N cells of i types with n_i_ of each type (! denoting the factorial operation) [27]. In normal tissues, there is a relatively small number of cell types within a given organ, each with similar function for a given class (e.g., epithelial, lymphoid, vascular). In cancer, new cell types emerge within the cancer mass, manifested as tumor cell heterogeneity (TCH). In order to appreciate the immense combinatorial complexity of biological systems, consider 1 gram of tissue with 10^9^ cells and only three different cell types. In this case K_c_ = 4.8 x10^8^ (since K_c_ is the logarithm of the combinations, the actual number of combinations is on the order of 10 followed by 100 million zeros). As the tissue volume and number of cancer and cancer-supporting cell types increases, global cancer combinatorial complexity increases further to astronomical levels. As with the three-body problem in physics, the problem can benefit by consideration and quantification of system symmetries and symmetry breaking. It is possible that combinatorial complexity mathematics could be applied as a measure of genomic TCH, leading to a new way to monitor how a tumor cell population is evolving and adapting over time. For example, what level of intervention is needed to effectively disrupt the cancer complexity and does combinatorial complexity increase or decrease after treatment with a therapeutic agent?

How does the cancer state of broken symmetry and a combinatorial complexity beyond that of normal tissues originate and evolve? A key hallmark of cancer is its ability to metastasize. It is thought that a key step in metastasis is the transformation of epithelial cells to mesenchymal cells [28]. Whereas epithelial cells may undergo malignant change and then grow within a tumor, these cells do not readily spread to distant sites in the organism. In contrast, when an epithelial cell is transformed to a mesenchymal cell the potential for cell spread outside the tissue of origin increases greatly. This is known as epithelial-to-mesenchymal transition or EMT. Mesenchymal cells have the physical configuration and other cellular machinery designed for movement. Known transcription factors and other cellular constituents are now recognized as key for maintaining a cell in the epithelial state, transformation to the mesenchymal state or conversion back to the epithelial cell type [28–30]. But what causes the molecular changes that then lead to cell symmetry breaking?

**Figure 1:**
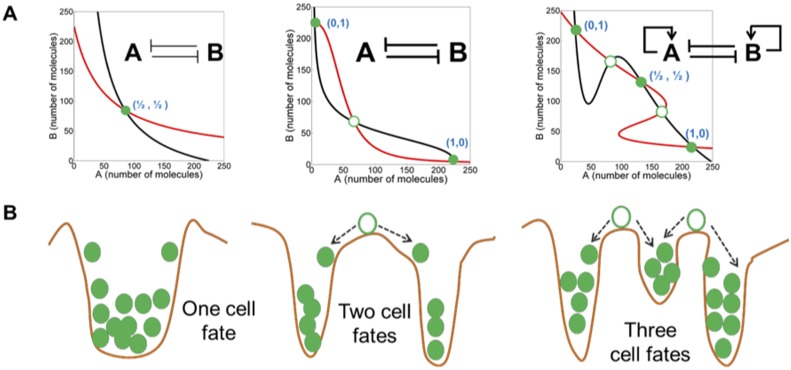
Symmetry breaking in mutually inhibitory feedback loops (from Jolly et al) Different levels of mutual inhibition and self-activation between two molecules, **A** and **B**, results in symmetry breaking from the configuration of equal numbers of A and B to bistability and tristability. In the tristable configuration, the intermediate state could represent a hybrid cell state. Red and black curves describe nullclines for A and B, and their intersections are the steady states. Green filled circles represent stable steady states, and green hollow circles show unstable steady states. For more information, see Jolly et al, 2015.

This question has recently been examined by the study of transcription factor and miRNA levels that together operate as a molecular switch to determine phenotypic cell fate. In this model, the transcription factors (e.g. ZEB, SLUG, and TWIST) and miRNA’s (e.g., miR-200 and miR-34) interact as auto-catalytic and inhibitory network components in a symmetry breaking decision network [31–33]. Small perturbations in one or more components can then cause a cell fate phase transition. High miR-200 and miR-34, low ZEB and SNAIL define the epithelial phenotype (E) and low miR-200 and miR-34, high ZEB and SNAIL result in the mesenchymal phenotype (M). Recent results suggest that intermediate levels of these cell constituents can result in a metastable E-M or partial EMT phenotype that, under specific environmental conditions or spontaneous stochastic cell fluctuations, can break symmetry into an E or M cell (Figure 1) [31, 34]. Of clinical significance is the observation that the symmetric hybrid E-M cell type in various cancers correlate with increased aggressiveness and metastatic characteristics [31, 34]. The hybrid E-M cells also are more prone to exist in the circulatory system as bound clusters of cells, which is thought to promote survival in the blood stream and eventual seeding of distant tissues [34]. Thus, symmetry retention or symmetry breaking of the E-M hybrid cell is likely a key factor in metastasis. Understanding of the underlying molecular events in stabilizing and transforming the E, M and E-M cell subtypes could lead to a better understanding of the metastatic process and permit control of these processes in cancer patients.

Tissue cells that lead to the creation of tumors and continually repopulate them with new cancer cells are known as Cancer Stem Cells (CSCs). Importantly, CSCs possess the traits to evade cancer therapeutics and to lie dormant for long time periods until they begin a process of high growth rate and a high evolution potential that gives rise to lethal phenotypic variation. Recent studies indicate that the E-M hybrid cell state is more likely to gain stemness properties [34, 35] and therefore to readily switch between invasive and proliferative modes to enhance survivability and correspondingly, lethality to the host. The search is now on to identify cell markers of stemness and E-M hybridicity. Network and molecular switch symmetry and symmetry breaking considerations could contribute to this effort by determining the conditions to break the E-M hybrid cell symmetry toward the E state, which has the least metastatic potential.

### Geometric symmetry breaking

Geometric symmetry breaking requires consideration of two modes: conventional geometric structures and fractal structures. Conventional symmetry breaking is routinely observed by pathologists in the daily diagnosis and characterization of cancer cells using light microscopy . Indeed, abnormal cell and nuclear shape is one of the most reliable diagnostic criteria for cancer and is closely related to prognosis [36–38]. The malignant potential of almost all cancers is based on the grading of abnormal nuclear structure by pathologists. Fractal structure in cancer is less well characterized, but is increasingly investigated as a diagnostic and prognostic indicator [39–42]. The loss of geometric self-similarity in cancer can occur at different spatial scales, from the structure of the plasma membrane to that of chromatin.

Geometric symmetry breaking can be utilized to characterize the molecular and biochemical processes that determine cell shape and how they are disrupted in cancer. A fundamental feature of cytoplasmic and nuclear composition is their viscoelastic composition. The viscous and elastic properties of the cell create a structure similar to a soft glass with power-law mechanical properties [43, 44]. The elastic components include the proteins actin and myosin and microtubules represent rigid structures. The presence of elastic components that can create tension together with the rigid microtubules provides for a tensegrity structure of the cell that maintains normal cell shape and is profoundly disrupted in cancer [43, 45, 46]. These structural elements have been collectively termed the tissue matrix (TM) system that is comprised of the extracellular matrix, the membrane matrix, the cytoskeleton, and the nuclear matrix. Conventional tensegrity structures from macro structures in buildings to cell-cell interactions to proteins to DNA consist of struts and cables under tension that can transmit mechanochemical information [43, 44, 47].

A basic property of tensegrity structures is stability [48–51]. Tensegrity stability is reflected in the structural behavior as the result of geometric deformation due to an external load. If the structure returns to its self-equilibrium configuration when the external load is released, then it is stable. Tensegrity structures possess symmetry properties in virtue of their geometric structure. This symmetry results in high stability to deforming forces and efficient information transfer that helps maintain homeostasis. In cancer, this stability is greatly diminished, as cancer evolves to increasingly malignant forms [52–55]. The extracellular cell matrix also possesses tensegrity properties, which, when disrupted, may also contribute to degraded information transfer from the environment and increase metastatic potential [43].

**Figure 2:**
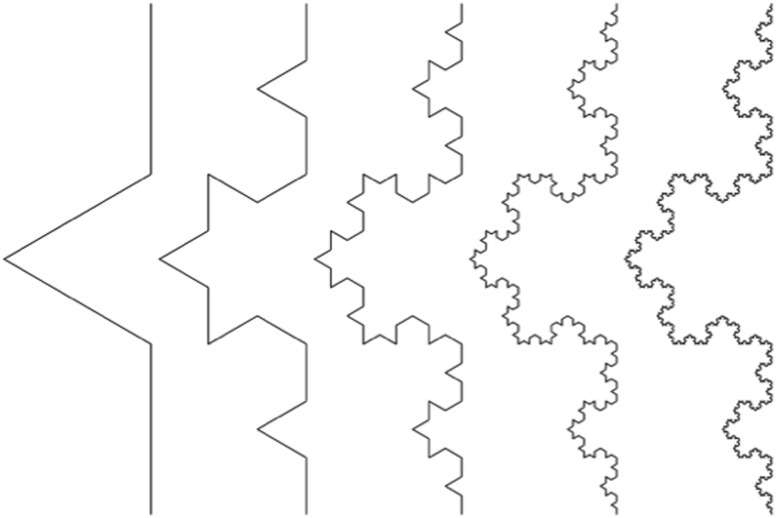
The Koch curve The Koch curve is created by dividing each line segment into thirds and replacing the middle segment with an equilateral triangle. Each iteration of the Koch curve produces a curve that is self-similar to the previous ones. It can be readily shown that the total length scales the power law relationship (4/3)n for n iterations and thus the length approaches infinity in the limit. The fractal dimension of a Koch curve is defined as log4/log3 = 1.2619. The Koch curve is continuous, but not differentiable, i.e., it has no tangent at any point.

Geometric complexity and symmetry can also be applied to the self-similarity of fractal structures, including down to the spatial level of DNA [41, 56, 57]. Fractals have a repeating geometrical description at any scale or spatial resolution. That is, there is no privileged spatial level of description, i.e., scale invariance exists. Conventional geometric structures obey integer scaling laws, for example the area of a square scaling as the power of 2 of the edge length. Fractal structures, however, display fractional scaling, such as the Koch curve whose length scales as the 4/3 power for each iteration [58] (Figure 2)

In fractal systems power laws describe the frequency of an occurrence x, as: f(x) = x^−n^. In this description, large events have a significantly greater likelihood of occurrence compared to, for example, when frequencies are distributed according to a normal distribution (i.e., “‘fat tails” exist). Thus, large-deviation phenomena in cancer may be related to fractal properties and power law behavior.

Fractal structures have been examined from the level of chromatin to the cell membrane to the lung and other organs [39–42, 59]. At the DNA level, the fractal structure of sequences can be examined in a ‘chaos game’ or in ‘DNA walks’, in which DNA sequences are examined to identify long-range correlations (as exist in physical phase transitions) in nucleotide sequences and disruptions represented by duplications, repeats and translocations [40]. In the chaos game a random walk over the DNA sequence space generates a fractal pattern. Using the 4 DNA base dimensionality, an information space pattern arises similar to the hyperspace and hypercycles of Eigen [60]. These analyses, then, are able to detect global gene structure and long-range pattern disruption in cancer that would otherwise remain opaque without considerations of self-similarity and power law dependencies. Thus, long-range correlations and symmetry breaking in gene structure could, in turn, affect integrated cell function and homeostasis as reflected in the loss of top-down and bottom-up global function. Fractal patterns also exist in functional networks, as addressed below [61].

Symmetry and symmetry breaking in the cancer cell have not yet been fully investigated, but this represents a fertile area for additional work in order to understand features of cancer cell shape and chemomechanical information transfer disruptions at a molecular level. In particular, it would be highly desirable to better understand the boundary limits of symmetry disruption in the progression of normal cell function to cancer to cell death. This could permit interventions to either further push the cancer state toward death or back toward normal homeostasis.

### Functional symmetry breaking

In functional complexity and symmetry breaking, the interactions among individual cellular constituents is examined [27]. These interactions form networks, such as protein-protein or gene-gene interaction networks. Individual networks don’t function in isolation, but interact with each other and build up a global system network that permits life. In cancer, the network is degraded and homeostasis is lost. Bioinformatics seeks to elucidate regularities and control points in biological networks that are lost in cancer, but as the previous combinatorial complexity examples demonstrate, the problem becomes even more computationally daunting when functional interactions are considered. Symmetry analysis can again contribute to clarifying this component of the cancer problem.

**Figure 3:**
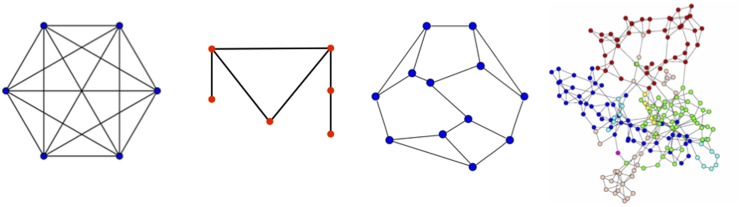
Graphs of differing symmetry Graph 1 is a complete graph with all nodes connected; it has 6 rotation and 12 reflection symmetries. Graph 2 is the smallest asymmetric graph. Graph 3 is the smallest asymmetric graph with each node possessing 3 edges (degree 3); it is known as the Frucht graph. Graph 4 is a complex graph, such as might exist for interacting proteins, for which any imbedded symmetries are difficult to discern by inspection. Software programs, such as nauty and SAUCY2, can compute the graph automorphisms.

The functional cell structure can be described by a network of interconnected individual molecular agents, which in turn can be analyzed by graph theory [62–64]. For example, in social networks, graphs show all of the connections between individuals. Graphs possess embedded symmetries that are important for stability and homeostasis [64–68]. In graph theory, the agents are the vertices and the connections are the edges of the graph (Figure 3). Graph theory entails a complex and exceedingly powerful mathematical framework that can identify symmetry and other features not readily discernable when the graph size reaches hundreds or thousands of independent agents. Two key graph theory concepts are graph symmetry and graph complexity, which are closely related, but provide different graph informational descriptions [62, 69–75]. Graph symmetry and symmetry breaking can characterize and explain changes in network functionality, complexity and information transfer. It is a foundational feature of a graph.

Graph symmetry is illustrated in Figure 3. The hexagonal graph with all vertices connected (a complete graph) is highly symmetric and include 6 rotations, 6 reflections along lines connecting opposite vertices and 6 reflections along lines connecting the midpoints of each edge. Any one or a combination of these symmetry operations maintains the network. In the second graph, there is no rotation, reflection or other change that maintains graph invariance; this graph is the simplest asymmetric planar graph. The third graph increases in complexity, but one can by careful inspection determine that the graph is asymmetric. This is the Frucht graph, which is the smallest asymmetric graph with each vertex having exactly 3 edges. In the fourth graph, the complexity is greatly increased compared to the three other graphs. A protein-protein interaction network might have this appearance. It is difficult to determine by inspection whether this graph has any symmetries.

In graph theory, a graph symmetry is termed a graph automorphism. The collection of all the graph automorphisms is the automorphism group or Aut(G). Measurement of Aut(G) and other graph properties lies on the cutting edge of computation theory and is a currently active area of research in the solution of very difficult problems [76–78]. Fortunately, new algorithms have made the calculation of Aut(G) much more practicable for large networks [79, 80].

The graph Aut(G) characterizes the information content of a functional network by counting all the symmetries. Yet, asymmetric components of a network can also store information and therefore parallel information approaches have been used for network analysis, including Shannon information, Kolmogorov complexity and Gibbs free energy [69, 71, 72, 74, 75, 81]. The Kolmogorov complexity (K(G)) measure is the size of the smallest computer program needed compute or construct the graph [69, 71, 82, 83]. A fully symmetric graph has a large Aut(G), low K(G) and low information. A completely asymmetric network has Aut(G) = 1 (a graph is symmetric with itself) and a K(G) that is a function of the actual size and arrangement of the individual agents, as is the information processing capacity of the network. Network symmetry and complexity measures are approximately reciprocal, but complexity measures also capture the information contained in asymmetric graph.

In recent years, the K(G) and Aut(G) analyses have been applied to a number of biological networks [64, 69–71, 81–88]. One advantage of Kolmogorov complexity over a purely symmetry description using Aut(G) is that it better captures all the graph structure by measuring all of the non-randomness. For a given graph Aut(G) variations in K(G) can be observed. Typically, there is an optimal number of edges for a given vertex number (V(G)) number to maximize K(G), and hence the information processing capacity of a network. For 50 nodes , K(G) is maximum at about 600 edges [83]. Related to this, is the phenomenon of phase transitions in complexity space when an abrupt increase in K(G) occurs at a threshold level of node connnetedness (i.e., the average node degree). This concept is similar to physical phase transitions where long-range interactions and symmetry breaking occur. When few connections exist at each node, K(G)~logV(G), but when the number of edges, E, grows, an abrupt increase in K(G) emerges at edge probability ~logV(G)/V(G) [69]. Is this the point where the phase transition from normal to cancer occurs or where a cancer suddenly develops resistance? Notably, the Aut(G) of protein interactions in different human cancers has been shown to be related to the 5-year survival rate [70]. Average symmetry may be sufficient for some of these analyses and would simplify the computational difficulty [89]. Further investigation could encompass the measurement of Aut(G) and subgroup symmetries in cancer and in the corresponding normal tissue in order to identify the precise location in protein, gene, or other networks where abnormal broken symmetries exist. To our knowledge, such experimental analysis has not yet been carried out. The identified sites could then be used as leverage points to attack and destroy the cancer network with molecular therapies or more challengingly, to restore the lost symmetries.

Biological networks must be stabilizable and controllable in order to store and transmit information necessary for survival of the organism. Survival entails a response to environmental changes, which in turn requires modification or fine-tuning of the cell or organism’s functional network over time. Homeostasis in dynamic, open systems far from equilibrium must exist at the interface between high order, maximum stability and a state of disordered chaos. As the size or number of nodes of a network (the graph order) increases the fraction of controllable systems decreases, thus permitting only a small percentage of complex systems to manifest homeostasis in response to external perturbations [90–93]. A second important concept from the theory of large networks is that a single leader-follower model is insufficient for controllability in complex systems [90, 92]. Thus, a multi-level consideration of top-down and bottom-up functionality is necessary for understanding an integrated system, such as would be required for recovering the controllability of cancer cells that existed prior to the neoplastic transformation. Cellular homeostasis has been recently examined with regard to singularities and stability against coordinate changes that preserve network structure [94]. This article advances our understanding of cellular homeostasis, but does not address symmetry, automorphism groups, or subgroup broken symmetries that could be related to attack tolerance when homeostasis is degraded; this could be a direction for future research.

Network symmetry considerations can contribute to understanding large network controllability and homeostasis. In recent years, the theory and application of network control principles has shown that network symmetry is closely related to controllability and stabilizability in response to external perturbations, decisions, and signals [91–93, 95]. Thus, the network Aut(G) could be a major determinant of controllability. In cancer, knowledge of broken symmetries that have a large effect on loss of homeostasis and creation of instabilities could provide new insights into tumor heterogeneity and metastatic spread. Early recognition of asymmetries could direct specific therapeutic interventions that could repair or reverse neoplastic processes and restore homeostasis or achieve a new homeostasis.

**Figure 4:**
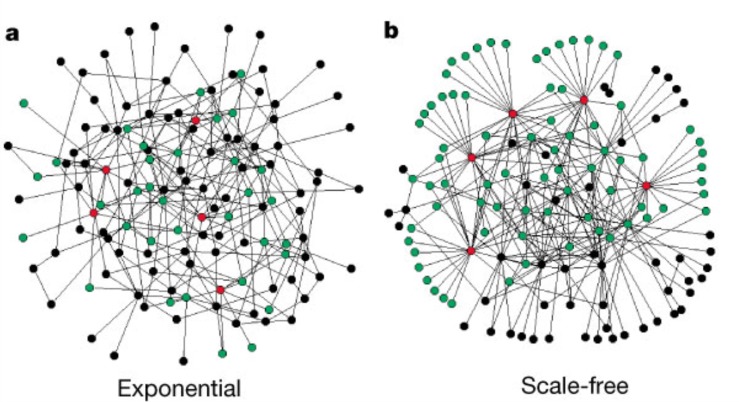
Visual illustration of the difference between an exponential and a scale-free network (from Albert et al) **a**, The exponential network is homogeneous: most nodes have approximately the same number of links. **b**, The scale-free network is inhomogeneous: the majority of the nodes have one or two links but a few nodes have a large number of links, guaranteeing that the system is fully connected. Red, the five nodes with the highest number of links; green, their first neighbours. Although in the exponential network only 27% of the nodes are reached by the five most connected nodes, in the scale-free network more than 60% are reached, demonstrating the importance of the connected nodes in the scale-free network Both networks contain 130 nodes and 215 links ((k)=3.3). For additional information see Albert et al, Nature 2000.

Another concept related to network symmetry is network attack tolerance [67, 96–102]. Attack tolerance is the network’s resilience to random or intentional deletion of nodes or interference with the connection of nodes (deletion of graph edges). Well-known examples are the WWW, power grids, and transportation networks. Among the network properties that determine robustness to attack, symmetry is a key factor [67, 96, 102]. As before, this is the case for the network symmetries defined by Aut(G), as well as the subgroup symmetries [62, 66, 67]. Scale-free symmetries that many real-world graphs possess also play a role in network resilience [61, 96, 97, 103] (Figure 4).

The relationship of network symmetry to cancer could take several different forms. For cancer origin, attack by environmental factors, such as carcinogens, with successful network disruption and conversion of homeostasis to instability could be investigated and better understood by application of symmetry principles, including my measuring the Aut(G) change after experimental application of carcinogens. In cancer treatment, understanding resistance to drugs or radiation could be enhanced though knowledge of attack vulnerability at specific points in the network, perhaps in the subgraphs that are asymmetric and have diminished attack tolerance. These new concepts will require improved elucidation of biologic network graph structure and improved analytics to detect imbedded symmetry groups.

## SUMMARY AND FUTURE RESEARCH DIRECTIONS

Symmetry and symmetry breaking concepts can define the parameters of cancer as a complex adaptive system. Defining symmetry in the physical sciences has been critical to the investigation of the structure of particles to the forces that define the universe. Biology is replete with symmetry and symmetry breaking events that are essential for life and evolution. In cancer, a further symmetry breaking occurs that disrupts the normal cell homeostasis to unleash a virulent and uncontrolled new life form that is often incompatible with host survival.

The three modes of symmetry breaking most commonly function in an integrated manner. For example, functional processes may interact with geometric changes in cancer and indeed, it may be that functional changes lead to the cytoskeletal tensegrity structural changes, each with its own symmetry group. Further examination of the three described modes of symmetry breaking as they apply to core features of cancer, origin, proliferation, metastasis and resistance, will be required. As these are defined for cancer cells to explain genomic and phenotypic diversity and plasticity, the observations and rules can be extended to include the tumor microenvironment and cancer at the tissue level. For example, can measures of system and molecular broken symmetry provide the needed information to determine sites with decreased attack tolerance for treatment? Could identification of broken cell symmetries permit repair in cancer in order to reestablish the lost homeostasis? Communication between the cancer and remote organs might also be better understood through functional symmetry considerations, as related to network stability, interference and attack tolerance, for example, by examining signaling networks from the cancer microenvironment to the metastatic niche of remote organs. Cancer destroys the host network at a system level and therefore, the cancer itself must be understood at its system level. Essential in these considerations is to not merely describe something as possessing a symmetry or broken symmetry, but to search for the molecular or system origin of the property and demonstrate how it can, in principle, be leveraged for the benefit of cancer patients.

### Memoriam

Don Coffey died on November 9, 2017 at 85. He was a tremendous inspiration and driving force for this article and a beloved friend. A remembrance for Don and the lasting contributions he made to cancer research and the careers of so many is included in this Issue.
